# Functions of tryptophan residues in EWGWS insert of *Plasmodium falciparum* enolase

**DOI:** 10.1002/2211-5463.12242

**Published:** 2017-06-05

**Authors:** Sneha Dutta, Anasuya Moitra, Debanjan Mukherjee, Gotam K. Jarori

**Affiliations:** ^1^ Department of Biological SciencesTata Institute of Fundamental ResearchMumbaiIndia; ^2^ Instituto de Medicina MolecularFaculdade de MedicinaUniversidade de LisboaPortugal; ^3^ Present address: T. H. Chan School of Public HealthGraduate School of Arts and SciencesHarvard UniversityBostonMAUSA

**Keywords:** dimer dissociation, moonlighting, *Plasmodium* Enolase

## Abstract

*Plasmodium falciparum* enolase (Pfeno) is a dimeric enzyme with multiple moonlighting functions. This enzyme is thus a potential target for anti‐malarial treatments. A unique feature of Pfeno is the presence of a pentapeptide insert ^104^ EWGWS ^108^. The functional role of tryptophan residues in this insert was investigated using site‐directed mutagenesis. Replacement of these two Trp residues with alanines (or lysines) resulted in a near complete loss of enolase activity and dissociation of the normal dimeric form into monomers. Molecular modeling indicated that ^340^R forms π‐cation bonds with the aromatic rings of ^105^W and ^46^Y. Mutation induced changes in the interactions among these three residues were presumably relayed to the inter‐subunit interface via a coil formed by ^46^Y : ^59^Y, resulting in the disruption of a salt bridge between ^11^R : ^425^E and a π‐cation interaction between ^11^R : ^59^Y. This led to a drop of ~ 4 kcal·mole^−1^ in the inter‐subunit docking energy in the mutant, causing a ~ 10^3^ fold decrease in affinity. Partial restoration of the inter‐subunit interactions led to reformation of dimers and also restored a significant fraction of the lost enzyme activity. These results suggested that the perturbations in the conformation of the surface loop containing the insert sequence were relayed to the interface region, causing dimer dissociation that, in turn, disrupted the enzyme's active site. Since *Plasmodium* enolase is a moonlighting protein with multiple parasite‐specific functions, it is likely that these functions may map on to the highly conserved unique insert region of this protein.

**Enzymes:**

Enolase(EC4.2.1.11).

**Abbreviations**
2‐PGA: 2‐phosphoglyceric acid
GST: glutathione S‐transferase
IPTG: isopropyl‐β‐d‐galactopyranoside
rPfeno: recombinant *P. falciparum* enolase
Tris: Tris‐(hydroxymethyl) aminomethane
W105,107A‐rPfeno: W105,107A variant of rPfeno
W105,107K‐rPfeno: W105,107K variant of rPfeno
Yeno: yeast enolase

Enolase (EC:4.2.1.11) is a highly conserved glycolytic enzyme that is largely present in the cytosol 1. Most enolases are dimeric barring a few bacterial ones that occur as octamers 2. Such oligomerization in non‐allosteric proteins invariably enhance the thermodynamic stability of the molecules 3. Recent observations from several different organisms have indicated that enolase is a multifaceted moonlighting protein that has a host of non‐glycolytic functions 4, 5, 6, 7, 8, 9, 10, 11. In *Plasmodium falciparum,* enolase is one of the highly expressed enzymes 12 that exhibits association with multiple subcellular compartments 4, 5. Interestingly, the pattern of distribution also showed stage‐dependent variation 4. The observed presence of enolase on the cell membrane of the three main invasive stages of the parasite i.e. sporozoites, merozoites 4 and ookinetes 11, 13 has stimulated intensive interest in determining its cell surface functions. Thus far, parasite enolase has been shown to act as a cell surface receptor for plasminogen and ligand for mosquito midgut epithelial receptor 14; a target for biliverdin 15; and a receptor for peritrophic matrix proteins 16, 17. Some of these biochemical activities are likely to be essential for host cell invasion as anti‐enolase antibodies neutralize the growth of the parasite in red blood cell stages, conferring partial protection against malaria 18, 19. Anti‐enolase antibodies also blocked ookinete traversal through the insect gut epithelium resulting in the failure of parasite transmission 11, 13, 14.

Complete sequencing of genomes from multiple species of *Plasmodia* had allowed for the deduction of size and structure of its respective proteomes. An interesting feature of the *Plasmodium* proteins is the presence of certain insert sequences that are usually of low complexity 20. In enolase, there is an insert of five amino acids ^104^EWGWS^108^ 21 that is evolutionarily conserved among all species of *Plasmodia*. Modeling of the 3D structure of Pfeno based on the crystal structure of *Toxoplasma gondii* 22, placed this insert sequence in a loop region on the surface of the molecule. In past, efforts were made for gaining insights into the functional significance of this insert sequence in apicomplexan parasites using deletion approach 23, 24. The idea was to delete the insert sequence, express the deletion variant protein and compare its properties with that of the wild type (WT). Some of the observed effects were (a) loss of most of the enzyme activity; (b) decrease in cofactor Mg(II) affinity; (c) poor thermal stability and (d) dissociation of the dimeric form into monomeric one 24. In order to pin down the biochemical function(s) of each of the amino acid residues, site‐directed mutagenesis was used to generate multiple variants. Replacement of Ser108 with Gly brought about a decrease in Mg(II) affinity but did not affect the oligomeric structure of the protein 25. Here, we report the effect of replacement of the two tryptophans (W105 and 107) in the insert on the kinetic and structural properties of rPfeno.

## Results

### Modeled 3D‐structure of *Plasmodium falciparum* enolase

**Figure 1:**
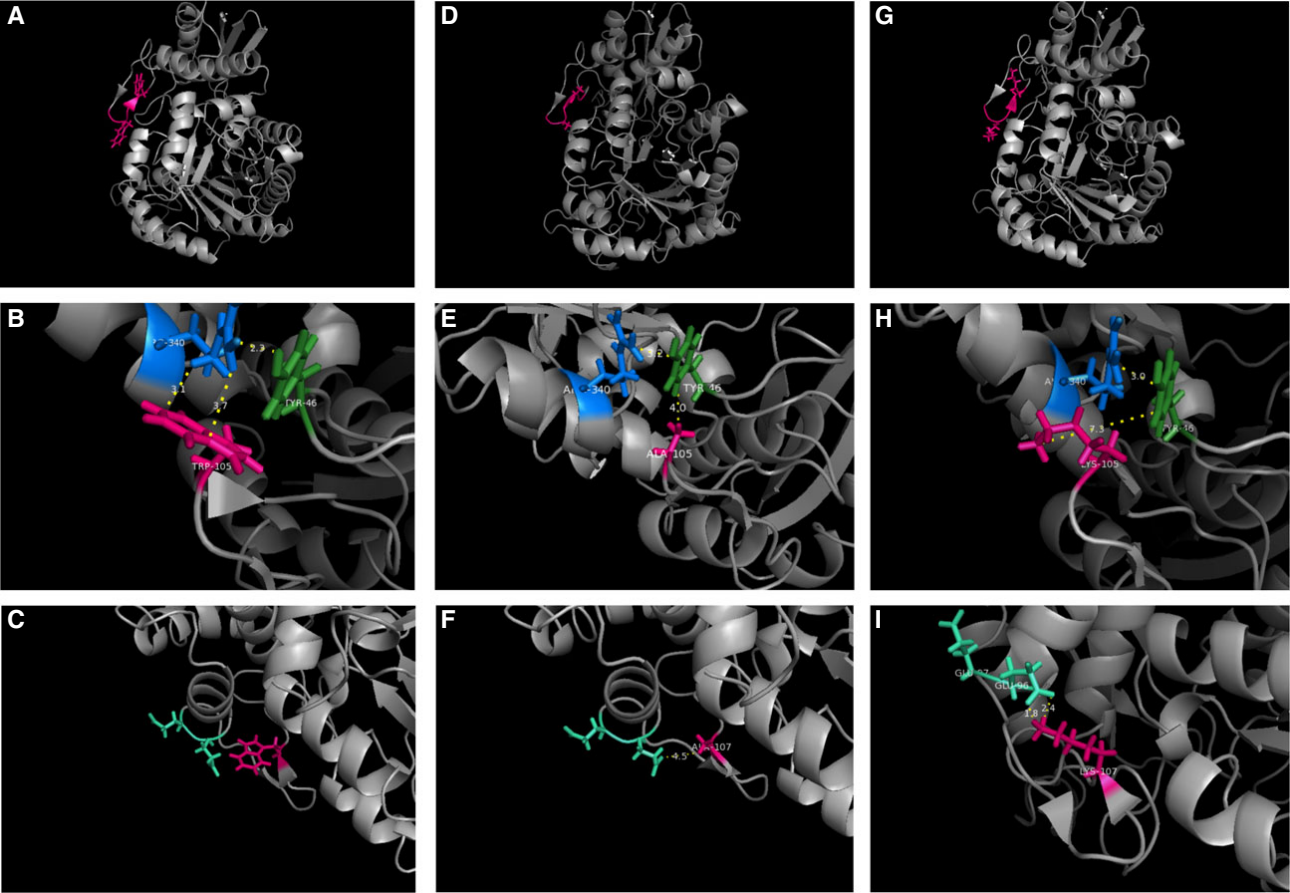
Modeled 3‐D structures of three different variants of Pfeno. Structures were generated using crystal structure of *Toxoplasma gondii* enolase1 (pdb: 3OTR) as template. Modeled 3D structure along with interactions of two tryptophan residues with neighboring regions are shown for all three variants. (A) WT Pfeno showing EWGWS (pink); (B & C) Interactions of W105 & 107 with neighboring residues; (D, E & F) Similar structures for W105,107A‐Pfeno and (G, H & I) for W105,107K‐Pfeno variants.

A model for the 3D structure of Pfeno was generated using homology based modeling tools and the X‐ray crystallographic structure of *T. gondii* enolase1 as a template (TgENO1; pdb: 3OTR) 22. Homology comparison between the enolases from *T. gondii* and *P. falciparum* showed 66.52% identity. The modeled structure of a monomeric Pfeno molecule is shown in Fig. 1A. The parasite specific insert sequence ^104^EWGWS^108^ is a part of a surface loop (marked in pink) with residues ^104^EW^105^ forming a β‐turn while ^106^GW^107^ formed a short anti‐parallel β‐sheet with the residues ^102^KN^103^. ^108^S is a part of a random coil structure and interacts with L^49^ that is located in a coiled segment formed by residues 42–50. Interactions of the side chains of the two tryptophan residues with the groups in neighboring region were also examined. The indole ring of W^105^ located in the turn region of the loop is in close proximity to the side chains of R^340^ and Y^46^. The two aromatic residues (W^105^ and Y^46^) are likely to have π‐cation interactions curtailing the rotational mobility of all the three residues (Fig. 1B). There was no interacting group in the vicinity of the indole ring of the distal W^107^ making it possible for the indole ring to have free segmental motion (Fig. 1C).

### Generation of Trp variants of rPfeno, over‐expression and purification

For investigating the functional significance of Trp residues in the insert sequence (W105 & 107) of Pfeno, following variants were generated (a) W105,107A [W105,107A variant of recombinant *P. falciparum* enolase (rPfeno)]; (b) W105,107K (W105,107K variant of rPfeno); (c) W105A,W107E and (d) EWGWS replaced with KAGAG as described in Materials and methods. The presence of the desired mutations was confirmed by DNA sequencing (Fig. S1).

**Figure 2:**
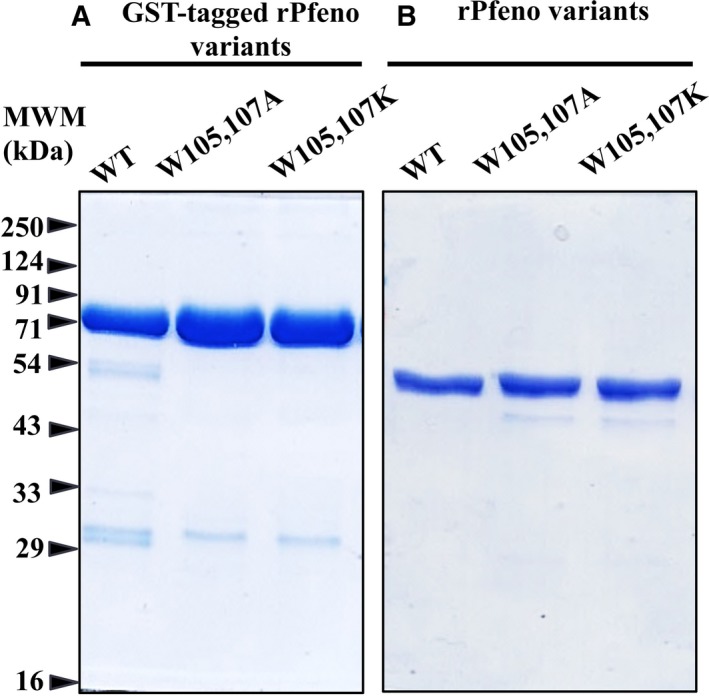
SDS/PAGE of purified (A) GST‐tagged WT‐rPfeno, W105,107A‐rPfeno and W105,107K‐rPfeno; (B) after the removal of the GST‐tag by proteolytic cleavage with thrombin.

For the preparation of variant proteins, multiple plasmids (pGEX4T1) harboring Pfeno variants were used to transform *Escherichia coli* BL21 (DE3) cells and cultures were checked for the over‐expression of the relevant proteins. All variants showed an over‐expressed band at ~ 75 kDa [expected MW for glutathione S‐transferase (GST) tagged‐rPfeno variants] upon induction with isopropyl‐β‐d‐galactopyranoside (IPTG; Fig. S2). The variant proteins were purified using affinity chromatography as described earlier 24, 25. Some of the variants (W105A, W107E‐rPfeno and KAGAG‐rPfeno) turned out to have rather poor solubility and did not stay in solution. Hence, these were not investigated any further. The remaining two GST‐tagged variants could be purified and the tag could be removed upon proteolytic cleavage by thrombin (Fig. 2). These preparations were > 95% pure as judged from the gel profiles.

### Protein sequencing using ESI‐LC‐MS/MS

**Figure 3:**
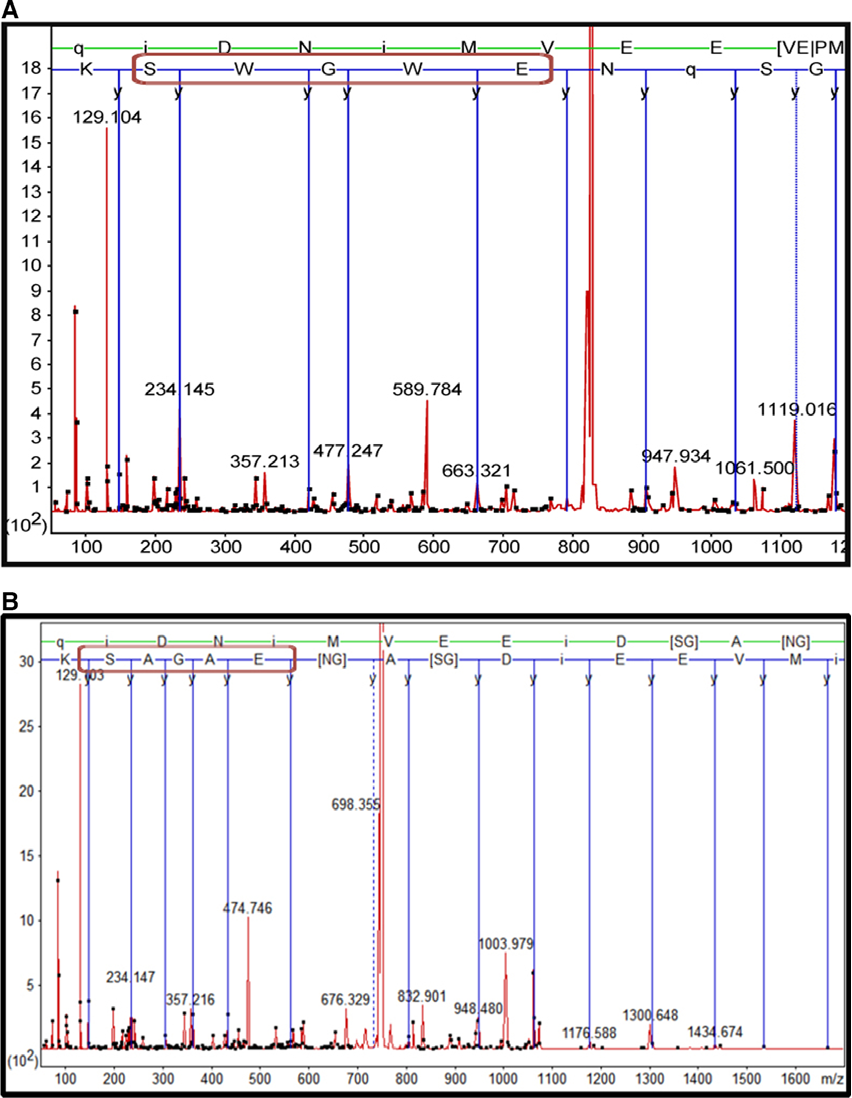
MS/MS spectra of tryptic peptides from (A) WT‐rPfeno and (B) W105,107A‐rPfeno. Only the relevant spectra corresponding to the sequence of the characteristic peptides EWGWS (WT‐rPfeno) and EAGAS (W105,107A‐rPfeno) are shown. MS/MS sequencing confirmed that both recombinant proteins have the changes made in the insert sequence.

For further confirmation whether the expressed variant proteins had the desired replacements, purified proteins were digested with trypsin and subjected to MS/MS analysis. One expects to get a tryptic peptide containing the EWGWS sequence that is characteristic of WT‐rPfeno and EAGAS sequence for W105,107A variant of rPfeno (W105,107A‐rPfeno). Since trypsin cuts at K/R residues, the characteristic sequence EKGKS for W105,107K‐rPfeno will get fragmented and will not be detectable in MS/MS sequencing. Figure 3 shows the MS/MS spectra of the relevant tryptic peptides sequenced in the analysis of digests derived from WT‐rPfeno and EAGAS‐rPfeno variant proteins. These observations confirmed that the relevant residues got replaced as desired.

### Effect of mutations on the secondary, tertiary and quaternary structures of the protein

**Figure 4:**
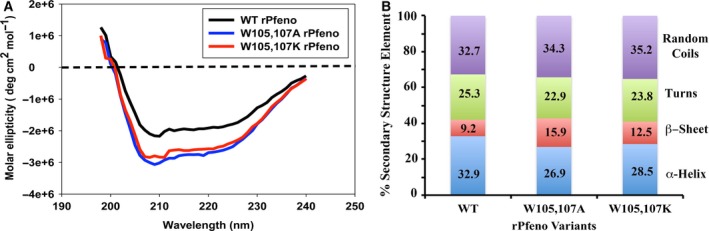
(A) Far‐UV CD spectra for WT‐rPfeno (black); W105,107A‐rPfeno (blue) and W105,107K‐rPfeno (red). Each spectrum was recorded using 0.5 mg·mL^−1^ protein in 50 mm Tris‐HCl, 150 mm NaCl and 5 mm MgCl_2_ pH 7.5. (B) Percent secondary structure elements. Quantitative analysis of spectra was carried out by Yang's method as described in Methods and materials.

Changes in the primary structure of a protein invariably lead to an effect on the higher order structures. Effect of replacement of W105 & 107 on the secondary structures of the two rPfeno variants was examined by measuring circular dichroism (CD) spectra (Fig. 4). These were analyzed using Yang's method 26 to obtain the fractions of various structural elements (Fig. 4B). As compared to the WT protein, a marginal increase in β‐sheet content with a concomitant decrease in α‐helical elements was observed for both the variants.

**Figure 5:**
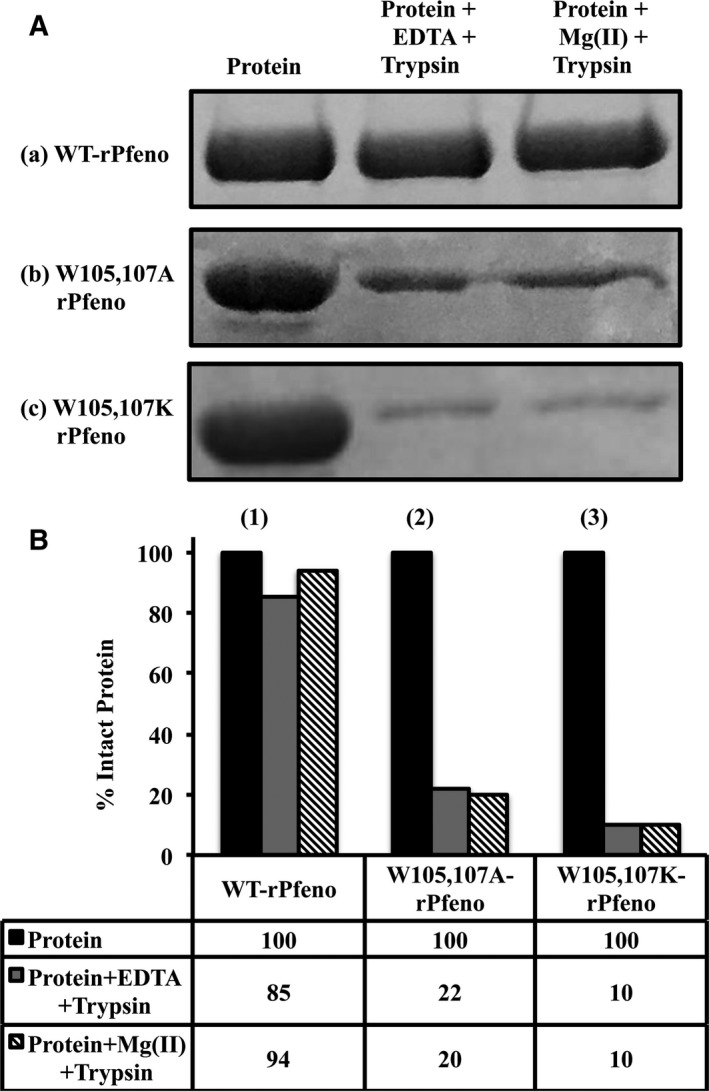
Limited trypsin digestion profiles for different variants of rPfeno. Purified proteins were subjected to trypsin digestion in presence and absence of Mg(II). (A) SDS/PAGE showing the intact proteins remaining after limited trypsin proteolysis. (a) WT‐rPfeno; (b) W105,107A‐rPfeno and (c) W105,107K‐rPfeno. Each sample contained 25 μg of protein in 100 μL of 50 mm Tris/HCl, pH 7.5. Lane1: total protein; lane 2: protein + 2 mm EDTA + 1 μg trypsin and lane 3: protein + 5 mm Mg(II) + 1 μg trypsin. Trypsin digestion was carried out at room temperature for 1 h and samples were analyzed on 12% SDS/PAGE. (B) Quantification of residual intact enolase protein in different samples.

Changes in tertiary structure were probed using an approach that provides a read out for the conformational state of a protein molecule. Limited trypsin digestion has been effectively used for such purposes 25, 27, 28, 29, 30. Most apo‐enolases from different organisms are known to exist in an ‘open’ conformational state (*E*_O_) that shifts to a ‘closed’ conformational state (*E*_C_) upon the binding of Mg(II) 31, 32. Since *E*_O_ is more susceptible to proteolysis as compared to *E*_C_ 25, the conformational states of apo and Mg(II) bound forms of W105,107A‐rPfeno and W105,107K‐rPfeno could be assessed by limited digestion with trypsin. In an experiment, the two Trp variants were subjected to limited trypsin treatment in the absence (with EDTA to ensure that the apo‐enolase was completely free of contaminating divalent cations) and the presence of Mg(II). After a short period of trypsin proteolysis, residual intact proteins in these samples were estimated using SDS/PAGE. Results are shown in Fig. 5A,B). WT‐rPfeno in its apo form is known to be in *E*_C_ form and is quite resistant to trypsin degradation. It did not show any significant reduction in the amount of intact protein and presence of Mg(II) had no effect on its susceptibility to proteolysis (Fig. 5A (a) and B). The variant proteins (W105,107A‐rPfeno and W105,107K‐rPfeno) where both tryptophan residues were replaced, showed high susceptibility to proteolytic degradation indicating that in apo‐form, both were in a conformation more akin to an *E*_O_ state. Further, in these mutants addition of Mg(II), also failed to convert *E*_O_ into *E*_C_ form (Fig. 5A (b,c) and B). These observations indicate that the replacement of W105 & 107 with A/K resulted in stabilization of apo‐enzyme in a state that is highly susceptible to trypsin digestion (similar to *E*_O_ state), but does not undergo Mg(II) induced conformational changes (unlike to *E*_O_ state). This inability of Mg(II) to induce essential structural changes for conversion of *E*_O_ like state into active *E*_C_ conformation would predict that both the mutant forms generated here would have very low or no catalytic activity.

**Figure 6:**
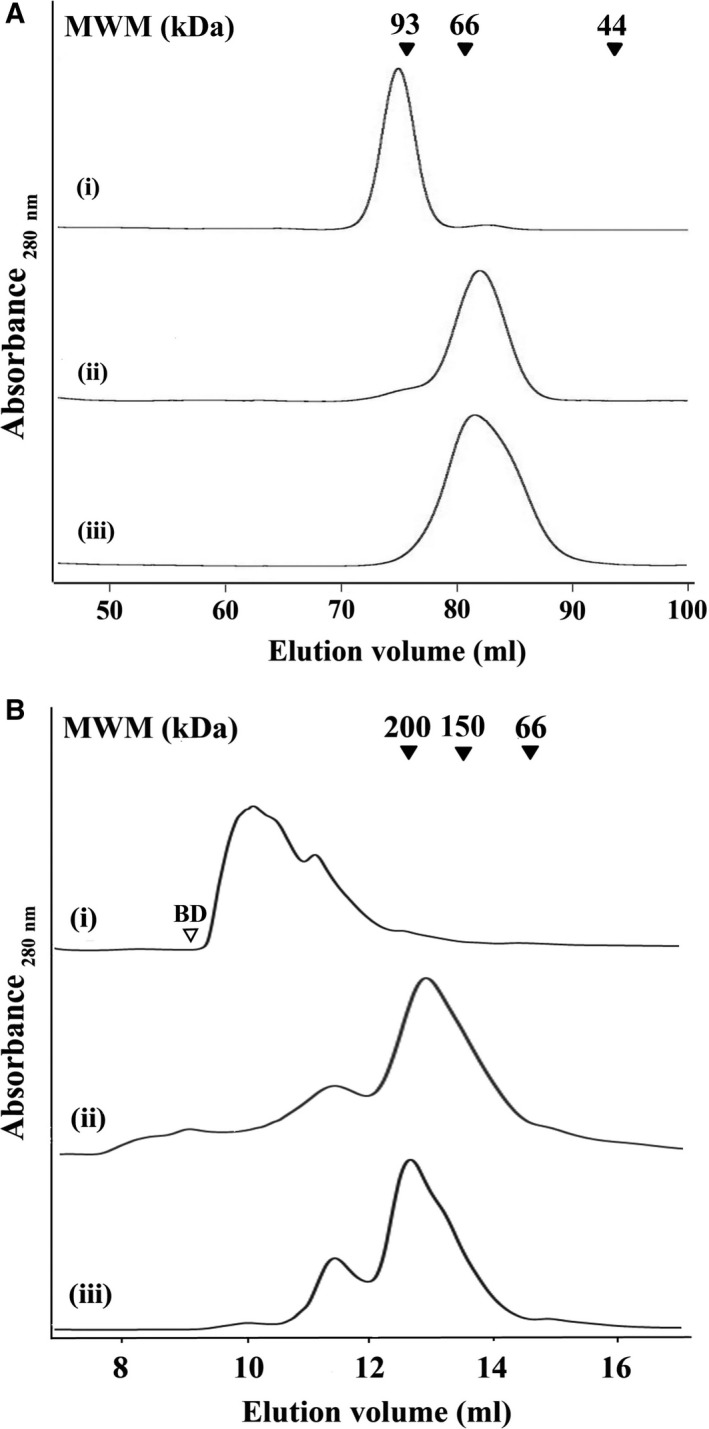
Size exclusion chromatographic profiles for (i) WT‐rPfeno; (ii) W105,107A‐rPfeno and (iii) W105,107K‐rPfeno. Variants were expressed and purified as GST‐tagged proteins. Oligomeric state was assessed (A) ‘without’ and (B) ‘with’ the GST tag. For the chromatographic runs, columns were pre‐equilibrated with 50 mm Tris‐HCl, 150 mm NaCl and 5 mm MgCl_2_ pH 7.5. (A) Superdex‐200 HiLoad 16/600 column was used with 1–2 mg of protein in 2 mL buffer and (B) Superdex‐200 Increase 10/300 GL column was used with 1 mg protein in 0.5 mL buffer.

For determining the effect of the amino acid replacements on the quaternary structure (i.e. oligomeric state) of the W105,107A‐rPfeno and W105,107K‐rPfeno, WT and the two variants of rPfeno were purified and then all three were subjected to size exclusion chromatography. The chromatograms for all three forms of enolase are presented in Fig. 6A. WT‐rPfeno eluted at ~ 100 kDa (dimer), while the two variants eluted at ~ 50 kDa (monomer). Thus, the replacement of two tryptophan residues in the insert sequence, that is located on the surface of the molecule, could disrupt the subunit‐subunit interactions at a distance of ~ 2.5 nm.

### Kinetic characteristics of W105,107A‐rPfeno and W105,107K‐rPfeno

**Figure 7:**
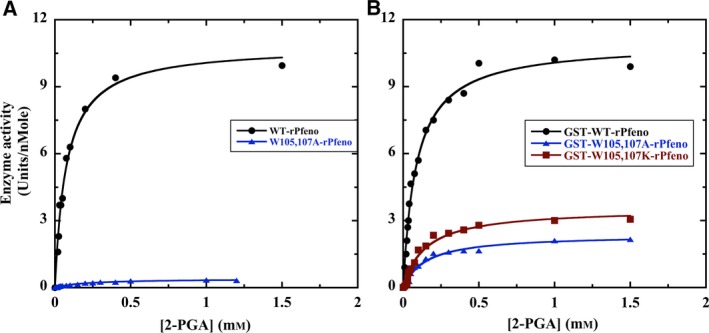
(A) Activities of WT‐rPfeno (black), W105,107A‐rPfeno (blue) and (B) activities of GST‐WT‐rPfeno (black), GST‐W105,107A‐rPfeno (blue) and GST‐W105,107K‐rPfeno (red) with increasing concentrations of 2‐PGA. Data were fitted to Michaelis‐Menten equation. The best‐fit values obtained for K_m_ (μm) and *V*_max_ (Units per nMole) for various forms of the enzyme were‐ WT‐rPfeno: *K*_m_ = 74 ± 7, *V*_max_ = 10.82 ± 0.36; W105,107A‐rPfeno: *K*_m_ = 208 ± 8, *V*_max_ = 0.40 ± 0.01; GST‐WT‐rPfeno: *K*_m_ = 87 ± 6, *V*_max_ = 10.95 ± 0.24; GST‐W105,107A‐rPfeno: *K*_m_ = 151 ± 18, *V*_max_ = 2.36 ± 0.09; GST‐W105,107K‐rPfeno: *K*_m_ = 147 ± 19, *V*_max_ = 3.53 ± 0.15. Assay mixture consisted of 500 μL of 50 mm Tris‐HCl, pH 7.5 with 1.5 mm Mg(II) and the substrate concentration was varied from 0.01 to 1.5 mm (10–1500 μm). For each assay 0.5 μg enzyme was used. Measurements were made in duplicate at 25 °C.

Effect of the replacement of the two Trp residues on the kinetic properties of rPfeno were assessed by measuring the initial rates of the reaction as a function of the substrate concentration and comparing these with WT‐rPfeno. Figure 7A shows the substrate concentration dependent changes in the enzyme activity for WT‐rPfeno and the W105,107A‐rPfeno. Equal amounts of protein were used in each assay (0.5 μg). These data were fitted to Michaelis‐Menten equation. Comparison of the best‐fit parameters for WT‐rPfeno and W105,107A‐rPfeno showed that the mutations brought about a three‐fold increase in *K*_m2PGA_ while the enzyme activity dropped by a factor of ~ 25. The W105,107K‐rPfeno variant did not show any activity even when ten fold excess protein (5 μg) was used for each enzyme assay indicating a complete loss of activity.

### Structural and kinetic characteristics of GST‐tagged forms of rPfeno variants

Replacement of tryptophans in the insert sequence resulted in substantial reduction in enzyme activity as well as in the affinity of inter‐subunit interactions leading to monomerization. Dissociation of native dimeric enolase into monomers is known to result in decreased enzyme activity by ~ 70% 33. Thus, the observed loss in enzyme activity upon replacement of Trp residues could arise either due to direct perturbation of active site and/or the mutation causing the monomer formation that in turn results in low activity. Essentially the two mechanisms differ as the first one assumes parallel changes in active site conformation and disruption of subunit interface interactions. The second mechanism proposes sequential process where primary effect of mutations is to dissociate the two subunits and that in turn leads to low activity as reported earlier for the WT 33. Dissociation of dimeric form in to monomers indicates lesser release of free energy on inter‐subunit interactions between the two mutants subunits. One way to shift the monomer‐dimer equilibrium in favor of dimer formation is to tag a surrogate protein that forms dimers to Pfeno. As GST is known to exist in dimeric form, we surmised that in the GST‐tagged forms of W105,107A‐rPfeno (or W105,107K‐rPfeno) the two mutated subunits will be held in close proximity raising their effective concentration. This could stabilize some of the inter‐subunit interactions that were adversely affected by mutations. To test this, GST‐tagged forms of all three variants of enolase were prepared and their oligomeric state and kinetic characteristics were assessed by gel filtration and enzyme activity measurements respectively.

The gel filtration chromatography profiles for the three GST‐tagged forms of rPfeno are shown in Fig. 6B. GST‐tagged WT‐rPfeno eluted in the void volume of the column (Fig. 6B (i)), while the two GST‐tagged tryptophan variants predominantly eluted as dimers at ~ 150 kDa (Fig. 6B (ii & iii)). As in GST‐tagged WT‐rPfeno, both domains (i.e. GST and r‐Pfeno) are capable of dimerization; they probably associate head to tail forming large aggregates as observed earlier 24. However, in the GST‐tagged W105,107A‐rPfeno (or GST‐tagged W105,107K‐rPfeno), only the GST domain had a dimerization capability and hence the oligomerization got restricted largely to dimers of ~ 150 kDa. To address the question whether such dimer stabilization also stabilized the active native structure of the mutant subunit, enzyme activity measurements were made on GST‐tagged forms of the two variants and compared with the activity of untagged forms (Fig. 7B). The best‐fit kinetic parameters obtained for different variants are presented in Table 1. The GST‐untagged form of W105,107A‐rPfeno had ~ 4% activity (of the WT) while the GST‐tagged form had ~ 22%. The activity enhancement effect was far more dramatic for the W105,107K‐rPfeno. The activity of untagged form was undetectable while coupling with GST restored the activity to ~ 33% of the WT. These observations imply that the contacts in the dimer interface of Pfeno contribute to the integrity of the active site structure. Further, tryptophan replacement with A/K brings about loss of inter‐subunit interactions resulting in dissociation followed by loss of activity. Stabilization of subunit‐subunit interactions also stabilizes active site conformation bringing about significant recovery in enzyme activity.

**Table 1:** Kinetic (*K*_m2PGA_ and *V*_max_) and the structural (oligomeric structure) properties of the variant proteins. *V*_max_ is expressed as % of WT‐rPfeno and for GST‐forms as % of GST‐rPfeno. Δ^5^‐rPfeno, pentapeptide ^104^EWGWS^108^ deleted rPfeno; ND, not detectable

S. No.	Enolase variants	*K*_m2PGA_ (μm)	*V*_max_ (% WT rPfeno)	Oligomeric state
1.	WT‐rPfeno^a^	74 ± 7	100	Dimer
	WT‐rPfeno (Imidazole)^c^	58 ± 12	39 ± 9	Monomer
	∆^5^‐rPfeno^a^	56 ± 10	1.0 ± 0.2	Monomer
	S108G‐rPfeno^b^	66 ± 13	50 ± 10	Dimer
	S108A‐rPfeno^b^	64 ± 6	40 ± 4	Monomer‐Dimer
	W105,107A‐rPfeno	208 ± 8	4.0 ± 0.1	Monomer
	W105,107K ‐rPfeno	ND	ND	Monomer
2.	GST‐WT‐rPfeno	87 ± 6	100	Higher oligomers
	GST‐S108G‐rPfeno	44 ± 5	66 ± 7	Higher oligomers
	GST‐S108A‐rPfeno	49 ± 3	64 ± 4	Higher oligomers
	GST‐W105,107A‐rPfeno	151 ± 18	22 ± 2	Dimer
	GST‐W105,107K‐rPfeno	147 ± 19	33 ± 4	Dimer
	GST‐∆^5^‐rPfeno^a^	68 ± 16	34 ± 4	Dimer

a Vora *et al*. 24.

b Pal‐Bhowmick *et al*. 33.

c Dutta *et al*. 25.

### Molecular modeling and inter‐subunit docking

Molecular modeling and subunit‐subunit docking could be used to visualize the structural changes that occur in the vicinity of the pentapeptide surface loop in W105,107A/K variants that possibly get transmitted to inter‐subunit interface. The monomer structure for each variant was modeled based on TgENO1 as described above and energy minimized. Relevant snapshots are presented in Fig. 1. Replacement of W^105^ with alanine led to loss of π‐cationic interaction between W^105^ and R^340^ resulting in to a displacement of Y^46^ as well as R^340^ (Fig. 1D,E). This is likely to enhance the conformational freedom for the pentapeptide loop. Substituting W^107^ with alanine did not seem to cause any change (Fig. 1E). The peptide region Y^46^‐Y^59^ that runs between the surface loop region and the subunit interface is likely to transmit the changes occurring at Y^46^. Structural changes that occur in the pentapeptide loop region in W105,107K‐rPfeno are of slightly different nature. Replacement of W105 with K eliminates the π‐cation interactions among R^340^, Y^46^ and W^105^ (Fig. 1H). Interestingly, the replacement of W^107^ by lysine creates a salt bridge between K^107^ and E^96^ as shown in Fig. 1I.

**Figure 8:**
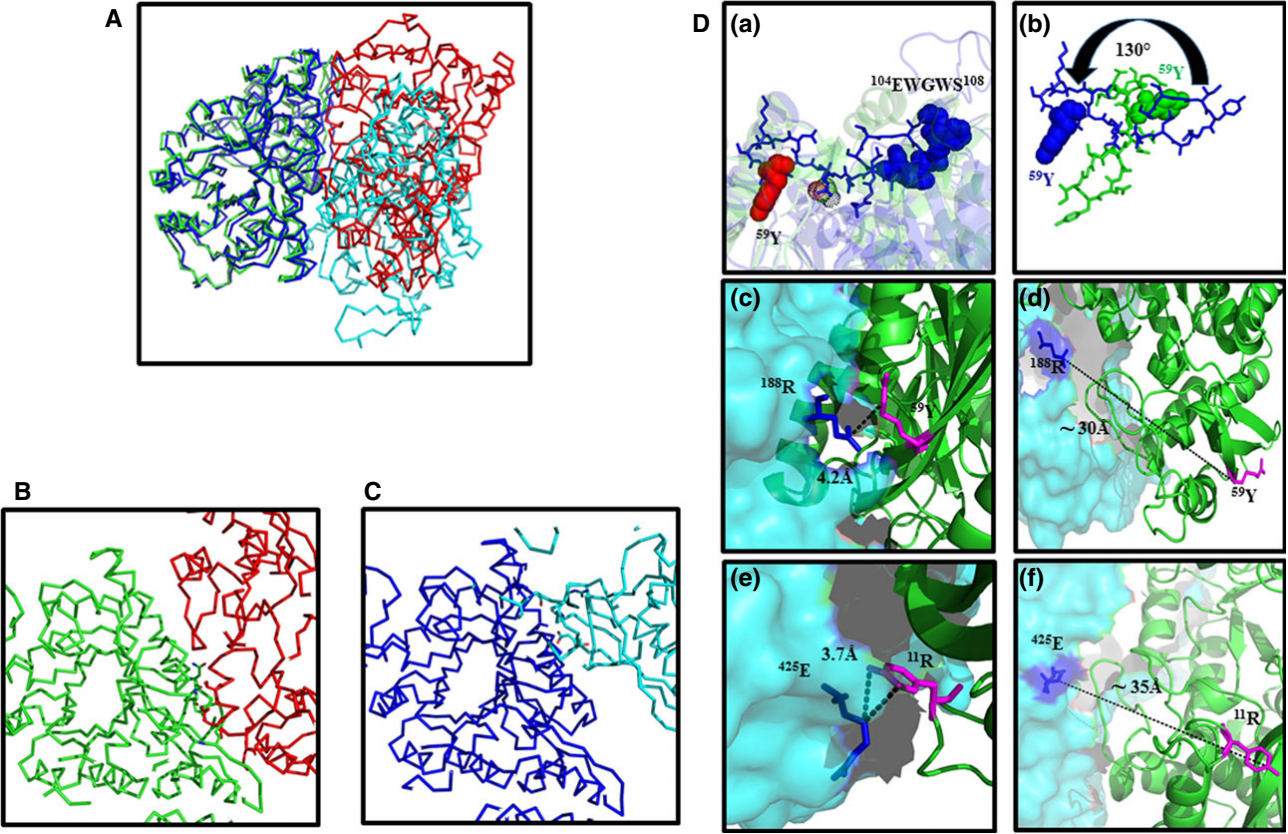
Subunit‐subunit interface interactions in docked dimeric structure of WT‐Pfeno and W105,107A‐Pfeno. Structure of each subunit was modeled as described in Fig. 1. Two identical subunits of WT or W105,107A‐Pfeno were subjected to docking using swarmdock software. (A) Overlay of WT dimeric Pfeno (green and red) and W105,107A‐Pfeno (blue and cyan). (B,C) A magnified view of the two interfaces. Different regions participate in inter‐subunit interaction in WT and alanine mutant. (D) Relative disposition of ^59^Y in two subunits of (a) WT and (b) W105,107A‐Pfeno. (c,e) Two key interactions between two native subunits that are suggested to stabilize dimer formation. (d,f) Absence of two interface interactions (i.e. ^188^R : ^59^Y and ^425^E : ^11^R) in W105,107A mutant form resulted in failure to form stable dimer.

In order to gain insight in dimer dissociation as a result of these mutations, we performed a subunit‐subunit docking experiment using SwarmDock (see Materials and methods). Two subunits of WT docked well with two critical inter‐subunit interactions namely a π‐cation bond between R^188^ : Y^59^ and a salt bridge between E^425^ : R^11^ as shown in Fig. 8D (c,e). Overlay of dimeric TgENO1 (pdb: 3OTR) and the modeled structure of Pfeno showed a RMSD of ~ 1 Å, indicating that the two monomers of WT‐Pfeno docked well (Fig. S3A). A close look at the inter‐subunit interface region of the two overlaid structures also showed a good overlap (Fig. S3B). Further, interface interactions between residues of two subunits for TgENO1 and Pfeno were also similar (Fig. S3C). Similar exercise of docking two subunits was also carried out for W105,107A‐Pfeno. The two subunits did establish a contact albeit in a different region of the molecule as compared to WT. Overlay of a native WT dimer (green & red) and alanine variant (blue and cyan) are shown in Fig. 8A. The two dimers differed in free energies (∆∆*G*) of their inter‐subunit interactions (i.e. ∆∆*G* ^o'^ = ∆*G* ^o'^_mutant_ − ∆*G* ^o'^_WT_) by ~ 4 kcal·mole^−1^. Figure 8B,C shows the subunit‐subunit contact surfaces in WT and W105,107A‐rPfeno. In the docked structure of the alanine variant, second subunit seemed to have flipped by ~ 130^°^ with respect to the WT position as indicated by the relative positions of Y^59^ of the second subunit in WT and Pfeno variant (Fig. 8D (b)). The RMSD for superimposed structures of dimeric WT‐Pfeno and W105,107A‐Pfeno was ~ 10 Å. This created a large separation between the two interacting pairs of R^188^ : Y^59^ and E^425^ : R^11^ in the variant dimer. Loss of such interactions resulted in a decrease of ~ 4 kcal·mole^−1^ in free energy of the W105,107A variant. This will correspond to an increase in *K*_d_ of the variant dimer by ~ 10^3^ folds as compared to the WT resulting in the dissociation of two subunits. Although, structurally there is a possibility for formation of a weak dimer in W105,107A‐Pfeno, under our experimental conditions of low protein concentrations (~ 2–3 mg·mL^−1^), no dimeric form was observable (Fig. 6A (ii)). Similar attempt to dock W105,107K‐Pfeno did not yield any dimeric structure indicating complete lack of any inter‐subunit interactions in this variant.

## Discussion

The *Plasmodium* proteome is peculiar in having numerous low complexity insert sequences 20. The functional significance of these elements is yet to be understood. *Plasmodium* spp. enolase has one distinct five amino acid insert with a sequence ^104^EWGWS^108^. Sequence and location of this insert is highly conserved among different species of the malarial parasite. In an earlier study, it was shown that the presence of this insert sequence stabilized the ‘Apo form’ of the parasite enolase in an active ‘closed’ conformational state 25. Deletion of this pentapeptide led to reduction in binding affinity of the cofactor Mg(II), substantial decrease in enzyme activity and dissociation of dimeric form in to monomers 24. For a detailed insight of structure‐activity relationship, a single residue replacement approach was taken. Replacement of S108 by glycine resulted in conversion of apo‐enolase from a closed active conformation to an open inactive state. S108G variant of rPfeno behaved much like other enolases that require Mg(II) induced conformational changes to acquire an active conformation. Further, this replacement did not affect the oligomeric state of the protein 25.

Experiments reported in this manuscript specifically dealt with the role of the two tryptophan (W105 & 107) residues in the insert sequence. The modeled structure showed these to be localized in a highly solvent accessible surface loop in Pfeno (Fig. 1). It is rather rare for hydrophobic tryptophan residues to be present on the molecular surface. Usually deviations from homology occur on the outer surface of protein molecules where changes are well tolerated without much of the structural consequences to the protein core. Structural characterization of the two variants (W105,107A‐rPfeno and W105,107K‐rPfeno) showed varying degrees of changes in their secondary and tertiary structures. There was a reduction in α‐helical content with a concomitant increase in β‐sheet in both the variants (Fig. 3). The effect on tertiary structure was evident from the substantial decrease in activity (Table 1; ~ 25 fold for W105,107A‐rPfeno and near complete loss for the W105,107K‐rPfeno). Further, the apo‐enzyme forms for both variants transitioned from an active *E*_C_ to an inactive open like conformation (*E*_O_ like). The *E*_O_ like conformation differed from *E*_O_ in lacking the ability to bind Mg(II) at the structural site and restore the fully active conformation as was observed in S108G variant 25. At the quaternary structure level, the dimeric protein dissociated to monomeric form (Fig. 6A). Thus, the replacement of the two tryptophan residues presumably located in a surface loop had drastic functional consequences. Trp variants completely mimicked the functional properties of the deletion variant. It is likely that the primary effect of the perturbation of Trp residues is to disrupt subunit‐subunit interactions, a long‐range allosteric type of effect since dimeric interface is ~ 2.5 nm away from the insert loop. Such dissociation in turn might have caused the destabilization of the active site conformation resulting in lower enzyme activity. Dissociation of native Pfeno [or yeast enolase (Yeno)] to monomers results in similar reduction in enzyme activity 33. These observations led us to predict that restoration of subunit‐subunit interactions could bring back the enzyme activity. To test this, we used a strategy where a GST tag was attached to the variants of Pfeno. Since GST forms a dimer, it will bring two subunits of Pfeno in close proximity facilitating the formation of dimers. If such interface interactions stabilize the active site, one would expect an increase in enzyme activity as compared to un‐tagged forms of the enolase variants. Data presented in Fig. 7 showed that attaching a GST tag to WT‐rPfeno did not have any effect on the activity. As WT‐rPfeno already has stable subunit‐subunit interface, it is anticipated that tagging with GST will have no effect on enzyme activity. However, the two mutated subunits when brought in close proximity in GST‐tagged form showed considerable increase in enzyme activity. The activity for W105,107A‐rPfeno increased by ~ 5 fold (blue curves in Fig. 7A,B). Increase in activity for W105,107K‐rPfeno was even more striking. In GST‐tagged form it had almost ~ 33% of WT activity while the untagged form was completely inactive (Table 1, Fig. 7).

In a recent study, tryptophan residues of the insert sequence were also implicated in interaction with certain PWWP domain proteins 17. As *Plasmodium* spp. enolase is known to be involved in multiple non‐glycolytic parasite specific functions 4, 11, 13, 19, 34, 35, it is quite likely that some of these moonlighting functions may map at this highly conserved unique insert sequence (EWGWS). Blocking this region (e.g. antibody) could result in perturbing some of the moonlighting functions that may be critical for the survival of the parasite. It will be interesting to test the functional consequences of anti‐EWGWS antibody treatment on the parasite growth and multiplication. It is clear from the modeled structure of Pfeno that none of the insert residues are a part of the active site or inter‐subunit interface. Thus, the effect of perturbation of insert residues is transmitted to the active site and dimer interface via the intervening structural elements. Further, the observed enhancement in the activity of mutant forms upon stabilization of dimeric interface indicated that allosteric communication prevails among the three regions viz. EWGWS insert, active site and subunit interface of the molecule.

If EWGWS insert sequence constitutes the site for protein‐protein interaction for performing moonlighting functions, it is likely that such interactions may result in subunit‐subunit dissociation and consequent loss of enzyme activity. This may provide a free subunit surface available for other molecules to interact for non‐catalytic functions. Switching off of one function to perform another is emerging to be a common feature of several moonlighting proteins 36, 37.

## Materials and methods

### Materials

All the chemicals used were of analytical grade. Sepharose beads coupled to glutathione were obtained from GE Healthcare (Uppsala, Sweden). Pfu polymerase, Pfu buffer and dNTP solutions were supplied by Thermo Scientific (Rockford, IL, USA). Primers were synthesized by Sigma Chemical Co (St. Louis, MO, USA). *Dpn*I was supplied by New England BioLabs (Ipswich, MA, USA). For the confirmation of the mutations to be at appropriate positions, BioInnovations performed the DNA sequencing. Markers used for size exclusion chromatography were obtained from GE Healthcare or Sigma Chem Co. Trypsin (sequencing grade) was purchased from Promega (Madison, WI, USA).

### Site‐directed mutagenesis

To replace Trp 105, 107 or other residues in *P. falciparum* enolase, whole plasmid mutagenesis method was used as described earlier 25. Plasmid pGEX4T1 harboring Pfeno 24 was used as a template. The primers used for substituting tryptophan with lysine/alanine/glutamate were

**Table:** 

Protein variant	Primer type	Sequence (5′–3′)
W105,107K‐rPfeno	Forward	GATGGAAGTAAAAATGAA**AAG**GGA**AAG**TCAAAAAGTAAATTAGG
	Reverse	CCTAATTTACTTTTTGA**CTT**TCC**CTT**TTCATTTTTACTTCCATC
W105,107A‐rPfeno	Forward	GGAAGTAAAAATGAA**GCC**GGA**GCC**TCAAAAAGTAAATTAGG
	Reverse	CCTAATTTACTTTTTGA**GGC**TCC**GGC**TTCATTTTTACTTCC
W105A,W107E‐rPfeno	Forward	GGAAGTAAAAATGAA**GCC**GGA**GAA**TCAAAAAGTAAATTAGG
	Reverse	CCTAATTTACTTTTTGA**TTC**TCC**GGC**TTCATTTTTACTTCC
^104^KAGAG^108^‐rPfeno	Forward	GGAAGTAAAAAT**AAAGCC**GGA**GCCGGA**AAAAGTAAATTAGG
	Reverse	CCTAATTTACTTTT**TCCGGC**TCC**GGCTTT**ATTTTTACTTCC

The codons used for mutated residues are marked in bold letters. Briefly, to obtain the desired mutations in rPfeno, complementary primers listed above were used. Typically, the reaction mixture had 1.5 μL of 10 mm forward and reverse primers, 0.5 μL of 10 mm dNTPs, 0.2 μL of proofreading Pfu polymerase (activity 2.5 U·μL^−1^), template DNA of 30 ng and 2.5 μL of Pfu buffer. The final volume was made up to 25 μL with autoclaved Milli‐Q water. Using an Eppendorf Thermocycler, the reaction mixture was subjected to PCR reaction. Conditions used for the reaction included 1.5 min of initial denaturation followed by 16 cycles of 30 s of denaturation at 95 °C; annealing for 1 min at 57 °C and an extension at 68 °C for 13.5 min. Final extension time used was 15 min. For the digestion of WT methylated parent strand of DNA, 1 μL of *Dpn*1 (20 000 U·mL^−1^) was added. Competent *E. coli* DH5α cells were transformed using 10 μL of Dpn I treated solution. Individual colonies were grown in LB medium and plasmid was isolated for each of the clones. DNA sequencing was performed by BioInnovations to confirm the mutations.

### Over‐expression and purification of different variants of Pfeno

pGEX4T1 plasmids harboring different variants were used to transform *E. coli* BL21 (DE3). Positive colonies were used for large‐scale culture (~ 1–2 L) and proteins were purified as described earlier 24, 25. Recombinant GST‐tagged form of various proteins was purified using glutathione Sepharose beads. Bead bound proteins were eluted either with glutathione (to obtain GST‐tagged form of the recombinant protein) or were subjected to thrombin (~ 1 U·mg^−1^ of recombinant protein) that cut the fusion protein, leaving the GST tag with the beads and releasing rPfeno variant in the supernatant. Supernatant was collected by centrifugation and thrombin was removed using benzamidine Sepharose beads. The purity of proteins was assessed by SDS/PAGE.

### ESI‐LC‐MS/MS

For peptide sequencing, purified variant proteins were digested with trypsin and analyzed by LC‐ESI‐MS/MS using an Agilent 6520‐QTOF mass spectrometer. The separation of peptides and MS/MS analysis was done as per previously published protocol 35.

### Gel‐filtration chromatography

Oligomeric state of the variants in GST‐tagged as well as untagged forms were determined using an Amersham‐Pharmacia Biotech AKTA‐FPLC system (Kwai Chung, Hong Kong) with Superdex‐200 HiLoad 16/600 or Superdex‐200 Increase 10/30 GL columns as described earlier 25. The molecular weight markers used were ovalbumin (44 kDa), bovine serum albumin (66 kDa), Yeno (93 kDa), alcohol dehydrogenase (150 kDa) and ß‐amylase (200 kDa).

### CD (circular dichroism) spectra

Circular dichroism spectra were recorded using Jasco J‐810 Spectropolarimeter (Tokyo, Japan). Typically a sample was placed in a quartz optical cell with 0.1 cm path length, 20 °C and the wavelength was scanned in the range of 180–260 nm. For each sample, three scans were averaged to improve S/N. The spectra were analyzed using Yang's method 26.

### Pulse proteolysis

Pulse proteolysis 38 technique was used to probe the tertiary structure as well as ligand induced conformational changes in different variants. Typically, ~ 25 μg of each variant protein in 100 μL of 50 mm Tris‐(hydroxymethyl) aminomethane (Tris)/HCl, pH 7.5 was subjected to limited proteolysis (1 h) using 1 μg trypsin at room temperature. Metal ion cofactor induced conformational changes were assessed as described earlier 25.

### Enzyme activity assays

Activity of enolase was measured in the forward direction by monitoring the conversion of 2‐phosphoglycericacid into phosphoenolpyruvate. The details for the measurements and assay conditions were the same as used earlier 25. A unit of enzyme activity was defined as the amount of enzyme that gave a change of OD of 1 au·min^−1^ under our assay conditions.

### Molecular modeling and Inter‐molecular docking analysis

3D‐structure of *P*. *falciparum* enolase (Pfeno) was modeled on the basis of the X‐ray crystallographic structure of *T. gondii* enolase1 (TgEno1; pdb: 3OTR) using the Automated Mode of Swiss Model PDB viewer 8.05 (http://swissmodel.expasy.org/). The amino acid sequence of Pfeno was obtained from UniProt/Swiss‐Prot database (Uniprot Accession No. Q27727; 446 amino acids). The optimized model structure was subjected to energy minimization using the software: MOE 2016.08 (Chemical Computing Group, Canada). The complex was prepared and parameterized using the Amber10: EHT force field. The system was energy minimized with a gradient RMS of 0.1 kcal·mol^−1^·Å^−2^ before starting the simulation. The modeled structures of the mutant version(s) of Pfeno were also generated using the same method as above.

For exploring the effect of mutations on oligomeric structure of Pfeno, rigid body docking between two monomers of Pfeno (modeled using TgEno1 structure as template; pdb: 3OTR) was performed using SwarmDock algorithm 39. In brief, SwarmDock uses a combination of local docking and particle swarm optimization to find in the docked complex low energy positions and orientations 40. Visualization of the structures was done using pymol 1.3 41.

## Author contributions

GKJ planned the research and analyzed data. SD and AM performed gene cloning, protein purification, structural and kinetic experiments. DM did the molecular modeling and docking analysis. GKJ wrote the manuscript. All authors read the manuscript and agree with the contents and conclusions described.

## Supporting information


**Fig. S1**. DNA sequencing chromatograms for the different variants of rPfeno confirming the presence of the desired mutations.

**Fig. S2**. Protein profiles of whole cell extracts derived from *Escherichia coli* BL21 (DE3) transformed with various plasmids expressing r‐Pfeno variants.

**Fig. S3**. Comparison of X‐ray structure of dimeric TgENO1 (pdb: 3OTR) and model of dimeric WT‐Pfeno.
