# Supplementary figures and images for: Functions of tryptophan residues in EWGWS insert of Plasmodium falciparum enolase
Source: FEBS Open Bio. 2017 Jun 5;7(7):892–904. doi: 10.1002/2211-5463.12242 (PMC5494301; doi:10.1002/2211-5463.12242)

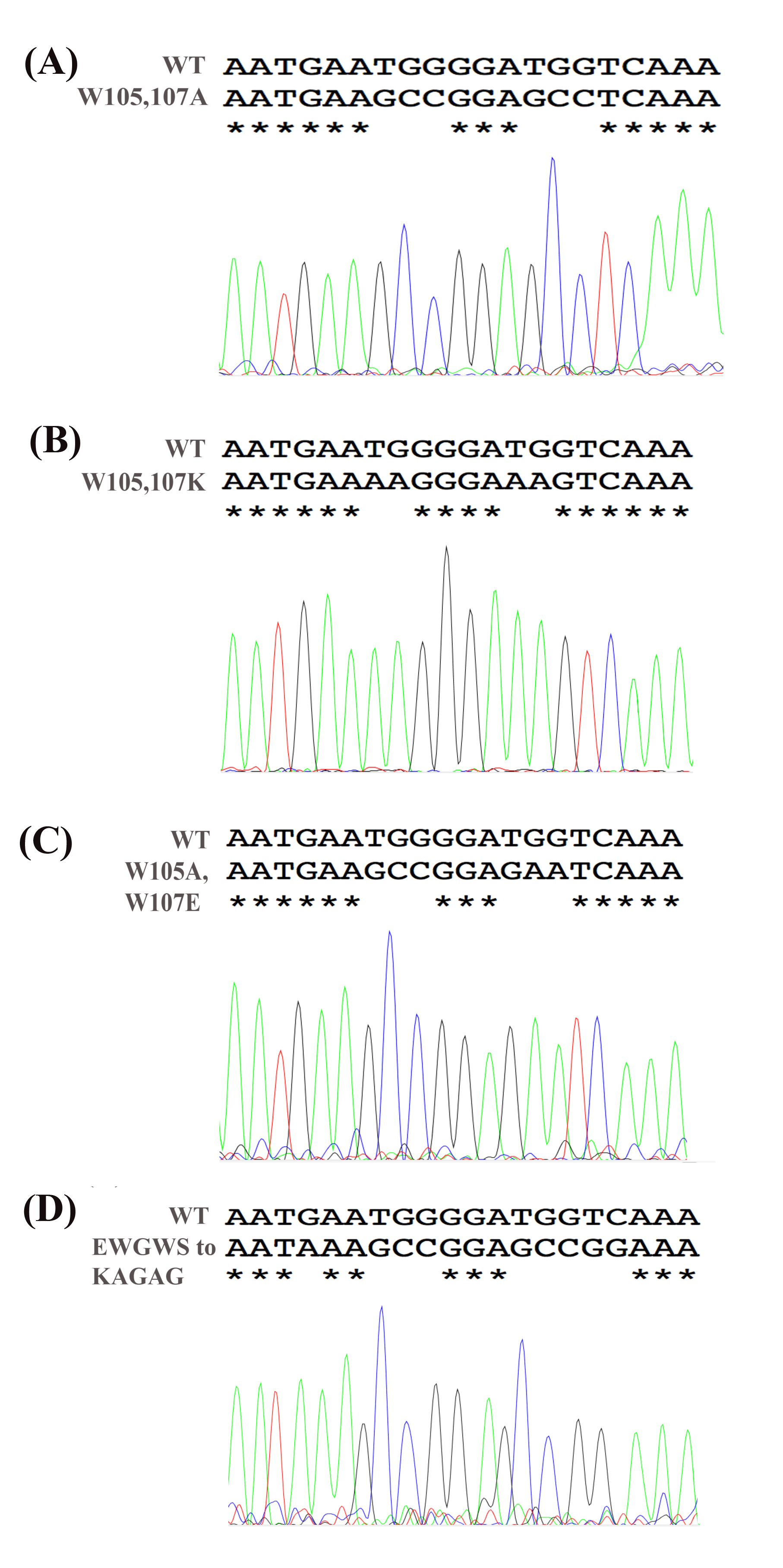

Supplement: Supplementary file 1 — Fig. S1. DNA sequencing chromatograms for the different variants of rPfeno confirming the presence of the desired mutations. [file FEB4-7-892-s001.jpg]

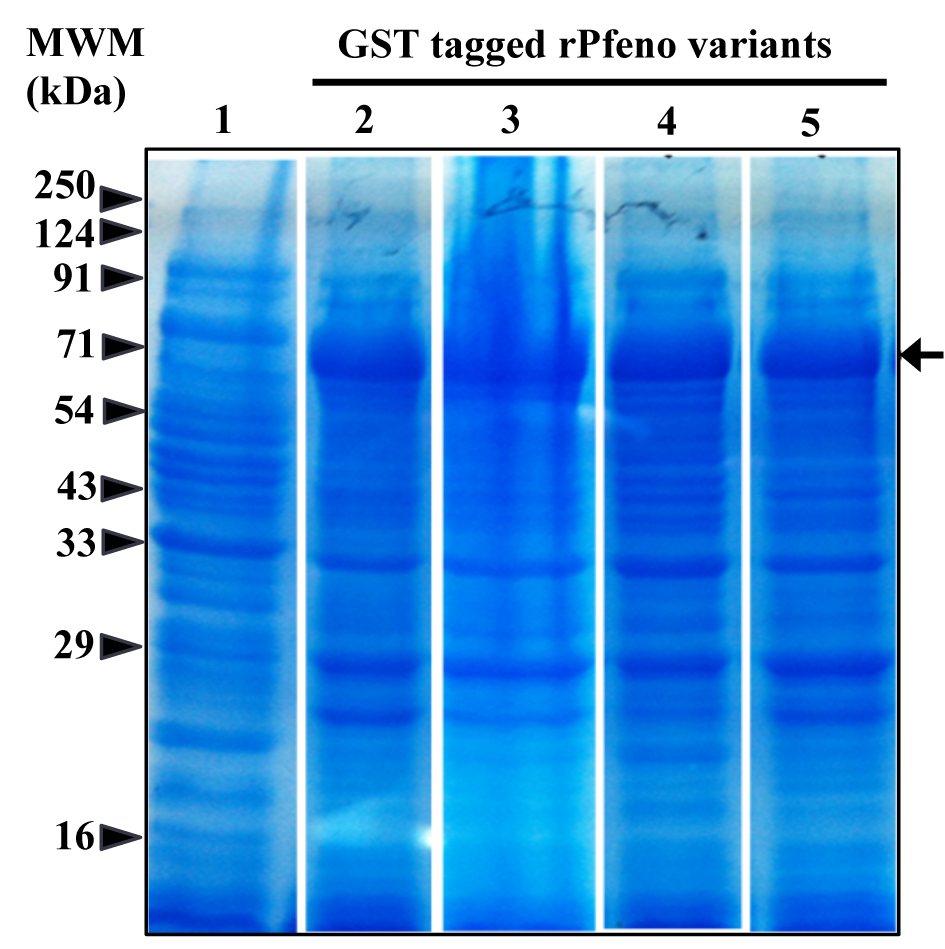

Supplement: Supplementary file 2 — Fig. S2. Protein profiles of whole cell extracts derived from Escherichia coli BL21 (DE3) transformed with various plasmids expressing r‐Pfeno variants. [file FEB4-7-892-s002.jpg]

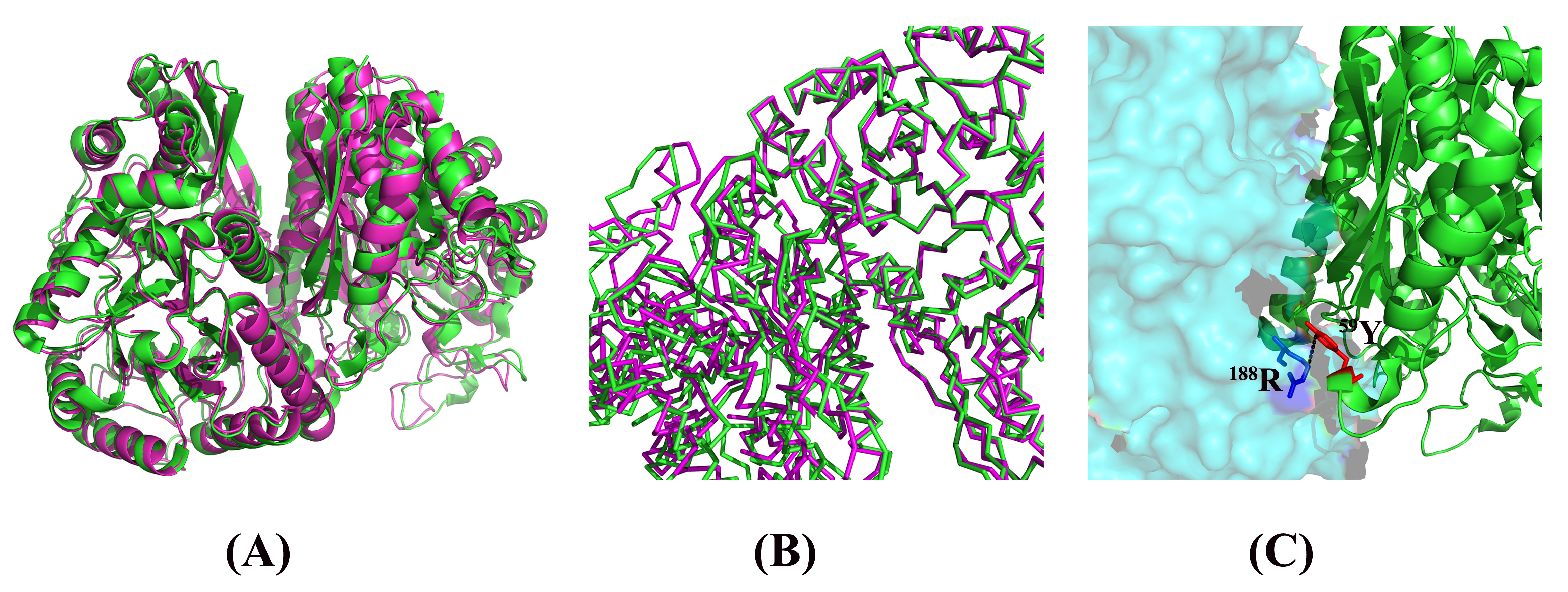

Supplement: Supplementary file 3 — Fig. S3. Comparison of X‐ray structure of dimeric TgENO1 (pdb: 3OTR) and model of dimeric WT‐Pfeno. [file FEB4-7-892-s003.jpg]
